# Metabolically Healthy Obesity is not a Myth

**DOI:** 10.1210/jcemcr/luad015

**Published:** 2023-02-24

**Authors:** Sarah S Farabi, Gordon I Smith, Jun Yoshino, Samuel Klein

**Affiliations:** Center for Human Nutrition, Washington University School of Medicine, St. Louis, MO 63110, USA; Office of Nursing Research, Goldfarb School of Nursing at Barnes-Jewish College, St. Louis, MO 63110, USA; Center for Human Nutrition, Washington University School of Medicine, St. Louis, MO 63110, USA; Center for Human Nutrition, Washington University School of Medicine, St. Louis, MO 63110, USA; Division of Nephrology, Endocrinology and Metabolism, Department of Internal Medicine, Keio University School of Medicine, Tokyo 160-0016, Japan; Center for Human Nutrition, Washington University School of Medicine, St. Louis, MO 63110, USA; Sansum Diabetes Research Institute, Santa Barbara, CA 93105, USA

**Keywords:** insulin sensitivity, weight gain, adiposity, case report

## Abstract

People with obesity who do not have the metabolic syndrome or components of the metabolic syndrome have been characterized as having metabolically healthy obesity (MHO). However, the existence of MHO has been questioned because people with MHO are at greater risk of developing diabetes and fatal cardiovascular disease than people who are lean and healthy. Here we report findings from a 25-year-old woman with rigorously defined MHO (normal oral glucose tolerance, insulin sensitivity [assessed using the hyperinsulinemic-euglycemic clamp procedure], plasma triglyceride, and intrahepatic triglyceride content) evaluated at baseline (body mass index, 37.7 kg/m^2^) and 5 years later, after a 32% (30.8 kg) increase in body mass (BMI, 49.6 kg/m^2^). Weight gain did not have adverse effects on fasting plasma glucose, oral glucose tolerance, β-cell function, insulin sensitivity, plasma triglyceride, intrahepatic triglyceride content, or carotid intima-media thickness. Adipose tissue expression of genes involved in extracellular matrix formation remained unchanged. Adipose tissue expression of several inflammation-related genes increased by more than 30%, but was not associated with a corresponding increase in plasma cytokine concentrations, with the exception of IL-6 and C-reactive protein. The present case study demonstrates that some people with obesity are resistant to the adverse cardiometabolic effects of excess adiposity and marked weight gain.

Obesity is typically associated with a variety of cardiometabolic comorbidities, including insulin resistance, atherogenic dyslipidemia, nonalcoholic fatty liver disease, prediabetes, and the metabolic syndrome [1]. However, some people with obesity do not have these complications and are considered “metabolically healthy” [2]. Even though the risk of all-cause mortality, type 2 diabetes, and coronary heart disease in people classified as having metabolically healthy obesity (MHO) is lower than those with metabolically unhealthy obesity (MUO), the risk of developing cardiometabolic comorbidities is still greater in people with MHO compared with people who are healthy and normal weight. In addition, many people with MHO convert to MUO over time [3]. As a result, it has been proposed that MHO does not really exist and all people with obesity are at increased risk for cardiometabolic diseases. A major limitation of these studies is the absence of a single, rigorous definition of MHO. More than 30 different definitions of MHO have been used in previous studies [4], and most criteria allow people to be classified as MHO even if they have evidence of metabolic abnormalities (eg, people with 1-2 metabolic syndrome components are still considered to have MHO in most previous studies) [5].

The possibility that there is a subset of people with obesity who are truly resistant to the adverse metabolic effects of excess body fat and weight gain is important because it provides a unique population to study the protective and pathogenic mechanisms involved in obesity-associated insulin resistance and metabolic diseases. Here, we present a unique case report that demonstrates MHO is not a myth.

## Case Presentation

A 25-year-old woman with MHO participating in an ongoing, longitudinal study was evaluated in July 2016 and again in October 2021. In 2016, the participant was considered to have MHO based on normal: (1) fasting glucose (<100 mg/dL [5.6 mmol/L]), oral glucose tolerance (2-hour glucose after ingesting 75 g glucose <140 mg/dL [7.8 mmol/L]) and hemoglobin A1c (HbA1c) (<5.7% [38.8 mmol/mol]); (2) plasma lipids (fasting triglyceride <95 mg/dL [1.1 mmol/L] and high-density lipoprotein (HDL)-cholesterol ≥50 mg/dL [1.3 mmol/L]); (3) intrahepatic triglyceride content (<5%) assessed by using magnetic resonance imaging; and (4) insulin sensitivity (glucose infusion rate > 8 mg/kg fat-free mass/min during a hyperinsulinemic-euglycemic clamp procedure) [5]. At enrollment, the participant did not have any medical illnesses other than obesity and was not taking any medications.

## Treatment

No interventions or therapies were provided between baseline studies in 2016 and follow-up testing in 2021.

## Outcome and Follow-up

Between 2016 and 2021, the participant gained 30.8 kg (32% increase) in body weight. The increase in weight comprised an 8.8-kg (20%) increase in fat-free mass, a 22.0-kg (42%) increase in total body fat, a 8.1-kg (37%) increase in leg fat mass assessed by dual x-ray absorptiometry, a 58% increase in subcutaneous abdominal adipose tissue volume, and a 78% increase in intra-abdominal adipose tissue volume assessed by using magnetic resonance imaging (Table 1). Intrahepatic triglyceride content also increased from 0.9% to 2.1%, but was still within the normal range.

**Table 1.** Participant characteristics before and after weight gain

	2016	2021	Normal reference range
**Body composition**			
Body mass index, kg/m^2^	37.7	49.6	—
Body weight, kg	97.6	128.4	—
Fat-free mass, kg	44.8	53.6	—
Body fat mass, kg	52.8	74.8	—
Body fat, %	54.1	58.3	—
Leg fat mass, kg	21.7	29.8	—
Subcutaneous abdominal adipose tissue, cm^3^	3790	5981	—
Intra-abdominal adipose tissue, cm^3^	631	1123	—
Intrahepatic triglyceride content, %	0.9	2.1	≤5.0
Blood pressure, mm Hg	127/84	128/85	<120/80
**Lipid profile**			
Triglyceride, mg/dL [mmol/L]	75 [0.85]	75 [0.85]	<150 [<1.7]
HDL-cholesterol, mg/dL [mmol/L]	52 [1.3]	32 [0.83]	>50 [<1.3]
LDL-cholesterol, mg/dL [mmol/L]	82 [2.1]	92 [2.4]	<130 [<3.4]
Total cholesterol, mg/dL [mmol/L]	149 [3.9]	139 [3.6]	<200 [<5.2]
**Glycemic control**			
Hemoglobin A1c, % [mmol/mol]	4.7 [27.9]	4.9 [30.1]	5.1-5.6 [32.2-37.7]
Fasting glucose, mg/dL [mmol/L]	90.0 [5.00]	88.6 [4.92]	64-99 [3.56-5.50]
Fasting insulin, mU/L [pmol/L]	24.7 [148.2]	16.7 [100.2]	2.6-24.9 [15.6-149.4]
OGTT 2-h glucose, mg/dL [mmol/L]	98.1 [5.45]	92.7 [5.15]	<140 [<7.78]
Basal ISR, pmol/min	339	326	—
β-cell function: ISR AUC_0-30_/glucose AUC_0-30_ (pmol × min)/(mmol/L × min)	155	158	—
Insulin sensitivity: GIR, mg/kg fat-free mass/min	8.2	9.8	—
**Plasma adipokines**			
Adiponectin, µg/mL	13.5	15.6	—
Leptin, ng/mL	129	190	—
**Liver biochemistries**			
ALT, IU/L	15	24	6-53
AST, IU/L	13	20	11-47
**Plasma inflammatory markers**			
PAI-1, ng/mL	15.5	10.5	—
CCL2, pg/mL	65.3	59.2	—
IFNγ, pg/mL	61.8	54.5	—
TNFα, pg/mL	8.4	7.3	—
IL-1β, pg/mL	1.1	1.0	—
IL-6, pg/mL	8.5	24.2	—
hs-CRP, mg/L	0.85	4.72	<3.0
**Marker of atherosclerosis**			
Carotid intima-media thickness*^a^*, cm	0.053	0.051	0.04-0.06

Abbreviations: ALT, alanine transaminase; AST, aspartate aminotransferase; AUC_0-30_, area under the curve during the first 30 min of OGTT; GIR, glucose infusion rate during a hyperinsulinemic-euglycemic clamp procedure; hs-CRP, high-sensitivity C-reactive protein, ISR, insulin secretion rate; OGTT, oral glucose tolerance test.

a Mean value of left and right common carotid arteries.

**Figure 1.**
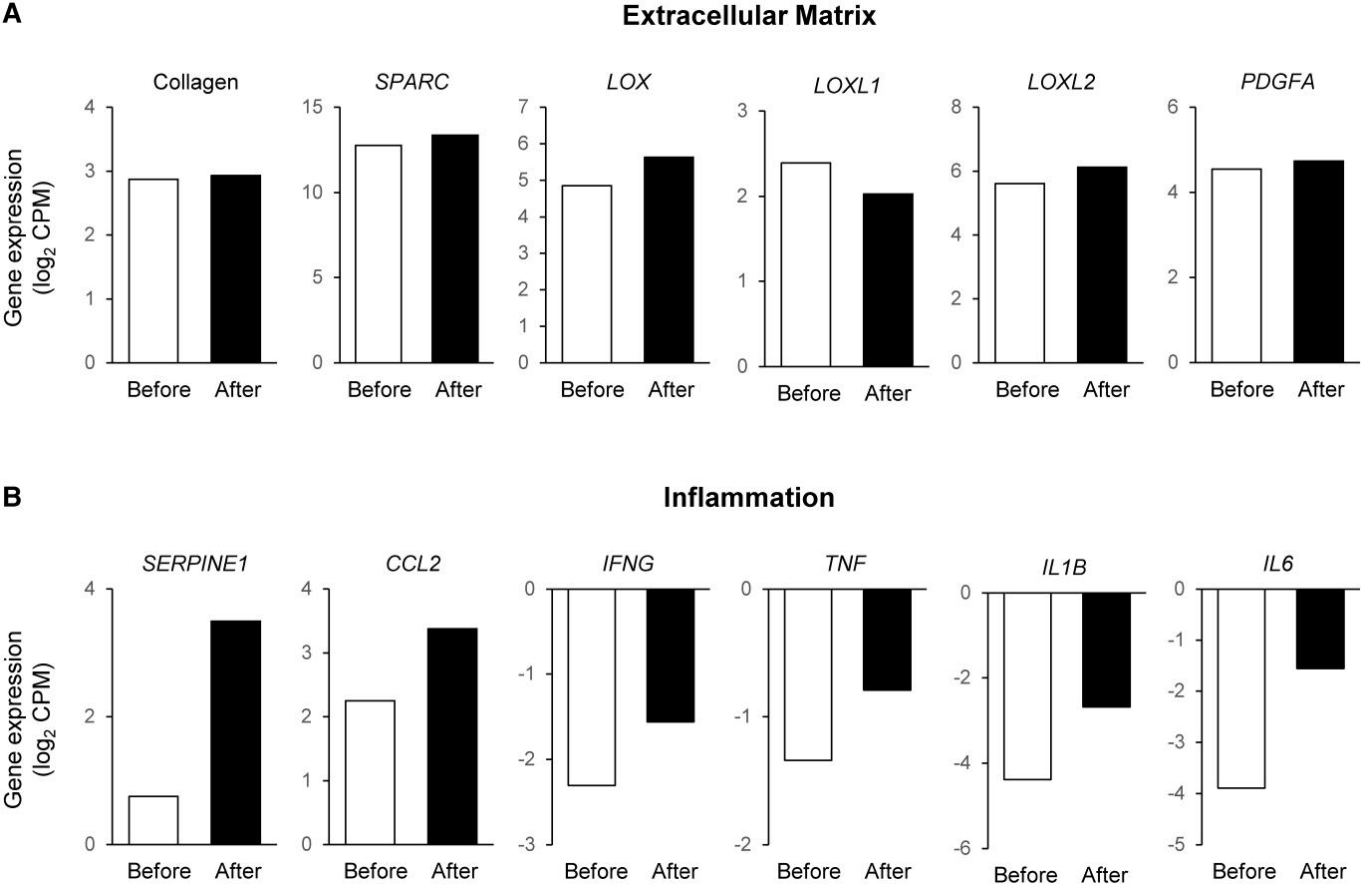
Subcutaneous abdominal adipose tissue gene expression before and after a 30.8-kg weight gain. (A) Expression of composite collagen genes (average of collagens 1A1, 1A2, 2A1, 3A1, 4A1. 4A2, 4A3. 4A4, 4A5, 4A6, 5A1, 5A2, 5A3, 6A1, 6A2, 6A3, 7A1, 8A1, 8A2, 9A1, 9A2, 9A3, 10A1, 11A1, and 11A2), key regulators of extracellular matrix remodeling (Secreted Protein, Acidic, Cysteine-Rich [Osteonectin; SPARC], Lysyl Oxidase [LOX], Lysyl Oxidase Like 1 [LOXL1], Lysyl Oxidase Like 2 [LOXL2], and Platelet-Derived Growth Factor Subunit A [PDGFA]) and (B) expression of genes encoding inflammation-related proteins (PAI-1, which is encoded by Serpine Family E member 1 [SERPINE1], CC motif chemokine ligand 2 [CCL2], interferon gamma [IFGN], tumor necrosis factor alpha [TNF], interleukin 1 β [IL1B], and interleukin 6 [IL6]). Expression of individual genes are presented as log_2_-transformed counts per million (CPM) reads.

Despite the marked increase in body weight and fat mass, there were no adverse effects on fasting plasma glucose, insulin, triglyceride, total cholesterol, low-density lipoprotein-cholesterol, and adiponectin concentrations, liver biochemistries (plasma alanine aminotransferase and aspartate aminotransferase concentrations), HbA1c, 2-hour plasma glucose, insulin sensitivity assessed by using the hyperinsulinemic-euglycemic clamp procedure, β-cell function (assessed as basal insulin secretion rate and insulin secretion rate in relation to plasma glucose concentration during the first 30 minutes after glucose ingestion), or carotid intima-media thickness (Table 1). The participant started taking an oral contraceptive (levonorgestrel/ethinyl estradiol biphasic extended cycle tablets) in 2016, which she continued through testing in 2021. No additional medication use or new medical illnesses were reported at follow-up. Plasma HDL-cholesterol concentration decreased from 52 to 31 mg/dL (1.34 to 0.80 mmol/L). However, this decrease was likely unrelated to weight gain because HDL-cholesterol decreased to 32 mg/dL (0.83 mmol/L) within 3 months of starting oral contraceptive therapy in 2016 without a change in body weight. The expression of genes involved in regulating extracellular matrix formation in subcutaneous abdominal adipose tissue, which are typically upregulated in people with insulin resistance and MUO [5], did not change, whereas the expression of genes that are considered markers of adipose tissue inflammation (*SERPINE1*, *CCL2*, *IFNG*, *TNF*, *IL1B*, and *IL6*) increased by more than 30% (Fig. 1). The increase in adipose tissue cytokine gene expression was not associated with a corresponding increase in plasma concentrations, with the exception of about a 3-fold increase in plasma IL-6 concentration. Plasma high-sensitivity C-reactive protein (hs-CRP) increased about 5-fold from 2016 to 2021 (Table 1).

## Discussion

In this case report, a woman with rigorously defined MHO remained metabolically healthy despite gaining nearly 31 kg (32%) in body weight over 5 years, resulting in an increase in body mass index from 37.7 to 49.6 kg/m^2^. Approximately 75% of the increase in body weight was due to an increase in body fat mass, including marked increases in leg, subcutaneous abdominal, and intra-abdominal adipose tissue depots. Although intrahepatic triglyceride content increased after weight gain, the value was still within the normal range found in metabolically healthy lean women. Many key measures of cardiometabolic function and disease risk, including glycemia (HbA1c, fasting blood glucose, and oral glucose tolerance test 2-hour glucose), factors that regulate glycemia (fasting insulin, β-cell function, and insulin sensitivity), plasma triglyceride, adiponectin, alanine aminotransferase and aspartate aminotransferase concentrations, and an index of atherosclerosis (carotid intima-media thickness) did not worsen and remained normal despite marked weight gain. The expression of adipose tissue genes involved in regulating extracellular matrix formation and collagen production, which are upregulated in people with MUO compared with those who have MHO [5] and are typically increased in association with weight gain-induced insulin resistance [6], did not change. The findings from this case report demonstrate a subset of people with obesity are resistant to the adverse cardiometabolic effects of excess adiposity and marked weight gain. The mechanism(s) responsible for this protective effect is not clear, but possibly involve adaptations in adipose tissue remodeling during expansion.

This case study supports the results from our previous study that found people with MHO were resistant to the adverse effects of moderate (∼6%) weight gain on insulin sensitivity and other measures of metabolic health [6]. Our current case report extends these findings and demonstrates that even extreme weight gain does not impair cardiometabolic function and health in a person who meets rigorous criteria for MHO. Although weight gain increased total and upper-body (subcutaneous abdominal and intra-abdominal) adipose tissue masses, which are commonly associated with an increase in insulin resistance and cardiometabolic abnormalities [5], these changes in body composition did not result in a deterioration in metabolic health in our participant. Moreover, there was no evidence of increased adipose tissue fibrosis (ie, increased expression of genes involved in extracellular matrix and collagen formation), which is typically associated with insulin resistance and MUO [5, 7]. This finding is consistent with the results from a study conducted in a rodent model that demonstrated preventing the fibrogenic adipose tissue response to marked weight gain prevented obesity-induced insulin resistance [7]. We also found the expression of several adipose tissue genes encoding proinflammatory cytokines increased after weight gain without a concomitant increase in most plasma cytokine concentrations. However, plasma IL-6 and hs-CRP increased 3- to 5-fold after weight gain. The effect of circulating IL-6 on metabolic function is unclear. Although IL-6 has been associated with insulin resistance, IL-6 infusion in healthy volunteers increases whole-body insulin sensitivity, assessed as insulin-stimulated glucose disposal during a hyperinsulinemic-euglycemic clamp procedure [8]. It is possible the increase in plasma hs-CRP concentration in our participant was caused by both an increase in plasma IL-6, which induces the hepatic synthesis of CRP [9], and her use of an oral contraceptive that increases plasma hs-CRP concentrations [10]. Nonetheless, the increases in plasma IL-6 and hs-CRP in our participant did have adverse effects on insulin action.

In conclusion, the present case study demonstrates that some people are resistant to the typical cardiometabolic abnormalities associated with obesity and marked weight gain. A better understanding of cellular and physiological mechanisms responsible for why some people do not develop obesity-induced cardiometabolic dysfunction could lead to novel therapeutic targets to prevent and treat obesity-related metabolic diseases.

## Learning Points

- There is a subset of people with obesity who are resistant to the adverse metabolic effects of excess adiposity.
- Marked weight gain in some people with obesity does not have adverse cardiometabolic effects.
- A better understanding of cellular and physiological mechanisms responsible for metabolically healthy obesity could lead to novel treatments for obesity-related cardiometabolic diseases.

## Data Availability

Some or all datasets generated during and/or analyzed during the current study are not publicly available but are available from the corresponding author on reasonable request.

## Abbreviations

HbA1c: hemoglobin A1c
HDL: high-density lipoprotein
hs-CRP: high-sensitivity C-reactive protein
MHO: metabolically healthy obesity
MUO: metabolically unhealthy obesity
